# Fecal Shedding of 2 Novel Live Attenuated Oral Poliovirus Type 2 Vaccine Candidates by Healthy Infants Administered Bivalent Oral Poliovirus Vaccine/Inactivated Poliovirus Vaccine: 2 Randomized Clinical Trials^[Author-notes jiab507-FM1]^

**DOI:** 10.1093/infdis/jiab507

**Published:** 2021-10-05

**Authors:** Christopher Gast, Ananda S Bandyopadhyay, Xavier Sáez-Llorens, Tirza De Leon, Rodrigo DeAntonio, José Jimeno, Gabriela Aguirre, Larin M McDuffie, Elizabeth Coffee, Demetrius L Mathis, M Steven Oberste, William C Weldon, Jennifer L Konopka-Anstadt, John Modlin, Novilia S Bachtiar, Alan Fix, John Konz, Ralf Clemens, Sue Ann Costa Clemens, Ricardo Rüttimann

**Affiliations:** PATH, Seattle, Washington, USA; Bill & Melinda Gates Foundation, Seattle, Washington, USA; Infectious Disease Department, Hospital del Niño “Dr José Renán Esquivel,” Panama City, Panama; Sistema Nacional de Investigación, Senacyt, Panama; Cevaxin, Panama City, Panama; Cevaxin, Panama City, Panama; VaxTrials, Panama City, Panama; Fighting Infectious Diseases in Emerging Countries, Miami, Florida, USA; Cherokee Nation Assurance, contracting agency to the Division of Viral Diseases, Centers for Disease Control and Prevention, Atlanta, Georgia, USA; Cherokee Nation Assurance, contracting agency to the Division of Viral Diseases, Centers for Disease Control and Prevention, Atlanta, Georgia, USA; Cherokee Nation Assurance, contracting agency to the Division of Viral Diseases, Centers for Disease Control and Prevention, Atlanta, Georgia, USA; Division of Viral Diseases, Centers for Disease Control and Prevention, Atlanta, Georgia, USA; Division of Viral Diseases, Centers for Disease Control and Prevention, Atlanta, Georgia, USA; Division of Viral Diseases, Centers for Disease Control and Prevention, Atlanta, Georgia, USA; Bill & Melinda Gates Foundation, Seattle, Washington, USA; PT Bio Farma, Bandung, Indonesia; PATH, Seattle, Washington, USA; PATH, Seattle, Washington, USA; Global Research in Infectious Diseases, Rio de Janeiro, Brazil; Institute for Global Health, University of Siena, Siena, Italy; Fighting Infectious Diseases in Emerging Countries, Miami, Florida, USA

**Keywords:** poliovirus, oral vaccine, viral shedding, nOPV2, infants

## Abstract

**Background:**

Primary intestinal immunity through viral replication of live oral vaccine is key to interrupt poliovirus transmission. We assessed viral fecal shedding from infants administered Sabin monovalent poliovirus type 2 vaccine (mOPV2) or low and high doses of 2 novel OPV2 (nOPV2) vaccine candidates.

**Methods:**

In 2 randomized clinical trials in Panama, a control mOPV2 study (October 2015 to April 2016) and nOPV2 study (September 2018 to October 2019), 18-week-old infants vaccinated with bivalent oral poliovirus vaccine/inactivated poliovirus vaccine received 1 or 2 study vaccinations 28 days apart. Stools were assessed for poliovirus RNA by polymerase chain reaction (PCR) and live virus by culture for 28 days postvaccination.

**Results:**

Shedding data were available from 621 initially reverse-transcription PCR–negative infants (91 mOPV2, 265 nOPV2-c1, 265 nOPV2-c2 recipients). Seven days after dose 1, 64.3% of mOPV2 recipients and 31.3%–48.5% of nOPV2 recipients across groups shed infectious type 2 virus. Respective rates 7 days after dose 2 decreased to 33.3% and 12.9%–22.7%, showing induction of intestinal immunity. Shedding of both nOPV2 candidates ceased at similar or faster rates than mOPV2.

**Conclusions:**

Viral shedding of either nOPV candidate was similar or decreased relative to mOPV2, and all vaccines showed indications that the vaccine virus was replicating sufficiently to induce primary intestinal mucosal immunity.

## Teaser

Fecal shedding of 2 novel oral poliovirus type 2 vaccine (OPV2) candidates and Sabin mOPV2 was assessed in bOPV/inactivated polio vaccine–immunized infants. Shedding of both candidates was similar or lower in quantity and duration compared with mOPV2. All 3 vaccines induced intestinal immunity.

Wild poliovirus types 2 and 3 have been eradicated globally [1], and circulation of wild-type poliovirus 1 is now restricted to Afghanistan and Pakistan [2]. A major obstacle to complete global eradication of poliomyelitis is the ongoing occurrence of outbreaks of circulating vaccine-derived polioviruses (cVDPV) originating from fecal excretion of live polioviruses administered as oral polio vaccines (OPVs) [3]. cVDPVs can lose key attenuations and reacquire neurovirulence during replication in vaccinees’ intestinal mucosa in settings of persistently low population immunity [4]. The WHO has re-organized it web site. This sentence should now read. In 2020 the World Health Organization (WHO) recorded 1107 cases of acute flaccid paralysis due to cVDPV [5], 1073 (97%) due to type 2 virus despite the global withdrawal of live type 2 virus in April–May 2016 [6].

Inactivated poliovirus vaccines (IPVs) cannot cause vaccine-induced paralytic poliomyelitis or VDPV but induce only marginal primary intestinal immunity, necessary to stop replication of live polioviruses on subsequent exposure, irrespective of the number of doses, amount of antigen, or route of administration [7, 8]. Therefore, cVDPV outbreak control relies on use of homotypic Sabin OPV vaccines to induce or boost intestinal immunity and interrupt transmission, although in rare circumstances using Sabin OPVs risks further “seeding” of new outbreaks. A decade-long joint-research project to meet the urgent public health need for more genetically stable OPV vaccines recently culminated in clinical assessment of 2 novel type 2 OPV (OPV2) candidates (nOPV2-c1 and nOPV2-c2) genetically engineered to be less likely to revert to neurovirulence [9, 10]. Clinical trials in adults, children, and infants demonstrated both candidates are safe, well tolerated, and immunogenic [11–13]. The candidate (nOPV2-c1) selected for further development was granted Emergency Use Listing (EUL) by the WHO for control of cVDPV2 outbreaks, the first vaccine so listed by the WHO, recognizing the risk of international spread of cVDPVs, which is designated as a Public Health Emergency of International Concern [14]. nOPV2 shedding in adults has been described [11, 12], but only preliminary results in children and infants have been reported [13]. We present the full evaluation of shedding in infants administered Sabin monovalent poliovirus type 2 vaccine (mOPV2) or the nOPV2 candidate vaccines. Understanding the kinetics of viral shedding in infants, the primary target age group for poliovirus outbreak response, is crucial to inform vaccination strategies to interrupt cVDPV2 transmission as well as to parameterize models of transmissibility.

## METHODS

We report stool viral shedding by infants originally recruited in 2 single-center, multisite, partially blinded, age de-escalation and dosage escalation randomized studies performed in the Cevaxin Vaccination and Research Center network, Panama [13]. The first study, from 23 October 2015 to 29 April 2016, was a prospectively designed “historical control” phase 4 study to provide baseline data with mOPV2 before its global withdrawal in May 2016 (ClinicalTrials.gov identifier NCT02521974). Panama had stopped using trivalent OPV (tOPV) containing type 2 in May 2014 in favor of the hexavalent diphtheria, tetanus, and whole-cell pertussis/hepatitis B/IPV/*Haemophilus influenzae* combination for routine infant immunizations but continued to use OPV for booster doses at 18 months and 4 years of age. The second study, performed in 4 Cevaxin centers from 19 September 2018 to 8 November 2019, assessed 2 nOPV2 candidates (ClinicalTrials.gov identifier NCT03554798). Both study protocols were approved by the Ethical Review Committee of the Hospital del Niño “Dr José Renán Esquivel.” Parents or guardians of all participants provided written informed consent. As previously reported [13], primary objectives were to assess the safety and immunogenicity of 2 nOPV2 vaccine candidates, compared with mOPV2 vaccine assessed in the historical study. The secondary objective, reported here, was to assess type 2 poliovirus shedding in selected stool samples.

### Participants

Eligible participants were healthy infants of either sex with birth weight >2500g enrolled at 6 weeks of age to ensure they received 3 doses of bivalent type 1 and 3 OPV (bOPV) at 6, 10, and 14 weeks of age and 1 dose of IPV at 14 weeks of age, at least 4 weeks prior to the first dose of study vaccine at 18–22 weeks of age. Infants who had not completed all these prior vaccinations were excluded. Other exclusion criteria included the presence of anyone in the infant’s household <6 months of age at the time of study vaccine administration, anyone who had received OPV within 3 months immediately before study vaccine administration, or anyone <10 years of age who did not have complete “age appropriate” poliovirus vaccination status at the time of study vaccine administration. “Age appropriate” was at least 3 doses of IPV for those <18 months, or at least 3 doses of IPV or tOPV plus 1 booster dose of any polio vaccine for those between 18 months and 10 years.

### Vaccines

mOPV2 used in the historical control study was Polio Sabin Mono Two (mOPV2), a Sabin strain type 2 (P712, Ch, 2ab strain) (GlaxoSmithKline Biologicals, Belgium, lot number DOP2A004AZ). Each dose of 2 drops (0.1mL) contained a nominal release dose of 10^5.7^ 50% cell culture infective dose (CCID_50_) of Sabin type 2 virus, administered using a supplied dropper.

Both nOPV2 vaccine candidates (nOPV2-c1 and nOPV2-c2), manufactured by PT Bio Farma (Jawa Barat, Indonesia), are attenuated serotype 2 polioviruses derived from a modified Sabin 2 infectious clone propagated in Vero cells, which include different combinations of 5 distinct modifications of the Sabin-2 genome [9, 10]. nOPV2 was administered as either a “low dose” in 2 drops (0.1mL) containing 10^5^ CCID_50_ using a supplied dropper, or a “high dose” containing 10^6^ CCID_50_ administered as 20 drops (1.0mL) from a syringe (or measured from a syringe into a spoon).

### Study Design

In the historical control study, all infants received 1 mOPV2 dose at 18 weeks of age; a randomly selected subset (n=50) received a second dose at 22 weeks. In the nOPV2 study, infants received 1 low (10^5^ CCID_50_) or high (10^6^ CCID_50_) dose of nOPV2-c1 or nOPV2-c2 at 18 weeks of age; randomly selected subsets (n=50) of each dose and candidate group received a second dose of nOPV2-c1 or nOPV2-c2 at 22 weeks.

### Viral Shedding

Stool samples collected daily for days 0–10 and 14, 21, and 28 after each vaccination (days 29–38, 42, 49, and 56 after a second dose) were processed for storage onsite at –20 °C for shipping to the Centers for Disease Control and Prevention (Atlanta, Georgia). Viral shedding was assessed using reverse-transcription polymerase chain reaction (RT-PCR) to detect viral RNA, and as infective virus in those positive for viral RNA [15]. Poliovirus genomes were detected using a Sabin multiplex real-time RT-PCR (rRT-PCR) assay of total nucleic acid extracted from stool suspensions (50%, w/v) using a KingFisher Flex 96-DW (Thermo Fisher Scientific, Waltham, Massachusetts) [16]. Before extraction, stool suspensions were spiked with an extraction control (Qβ bacteriophage) (Attostar, Edina, Minnesota) detected using a Qβ-specific rRT-PCR; stool suspensions with a negative extraction control (cycle threshold>40 indicating inefficient extraction) were reextracted. At fixed time-points, infectious virus was titered from subsets of stool samples that were PCR-positive for type 2 virus alone, using a modification of the WHO cell sensitivity assay and measured as the CCID_50_ per gram of stool (titer) [17].

### Statistical Analysis

Sample sizes were chosen to provide adequate data for the primary safety and immunogenicity objectives. All participants correctly receiving the respective dose of study vaccine were evaluable for viral shedding; those with poliovirus type 2 RNA detected in any prevaccination stool were excluded from the analyses. Samples with RT-PCR–detected poliovirus types 1 or 3 in addition to type 2 were not evaluable in the infectivity assay and are considered missing. All samples were evaluated via RT-PCR and if positive for type 2 alone, also for viral titer (log_10_ CCID_50_/g), except after the second dose when CCID_50_/g was evaluated only for days 5, 7, and 14 following nOPV2-c1 vaccination.

Summaries of the detection of vaccine virus via RT-PCR and viral titer were computed by group and time point. A shedding index endpoint (SIE) was calculated as the average titer of samples collected at 7 (±1), 14 (±3), 21 (±2), and 28 (±2) days after vaccination, with the lower limit of quantitation (2.75 log_10_) contributing as an observed value, and with RT-PCR–negative values contributing 0 to the mean [15]. The area under the curve (AUC) of shed virus was computed on the CCID_50_ scale via the trapezoidal rule, then log_10_-transformed for analysis.

Comparisons are presented of post-first-dose data, with both 1- and 2-dose groups contributing, showing proportions of each group with a PCR-positive sample at each time point. Comparisons are also made among infants who received second doses of mOPV2 or either of the nOPV2 candidates. Shedding rates were computed with exact confidence intervals (CIs), with comparisons conducted with Fisher exact test; log_10_ CCID_50_/g results, including indices, were summarized using the median, accompanied by bootstrap-based CIs, with comparisons conducted via the difference in medians and accompanied by the Wilcoxon test *P* value. Time to cessation of viral shedding was computed using Kaplan–Meier methods with comparisons performed via the log-rank test. Definitions of cessation included the first of 3 consecutive negative RT-PCR samples (time to shedding cessation), and time to culture negativity and time to transmission negativity, defined as the day of the last sample exceeding 2.75 log_10_ CCID_50_/g and 4.0 log_10_ CCID_50_/g, respectively. The value 4.0 log_10_ CCID_50_/g is used as a risk analysis threshold for reduced risk of transmission for Sabin OPV strains [18]. Subjects who did not shed or for whom cessation was not observed were considered right-censored. SAS version 9.4 software was used for analyses.

## RESULTS

### Demographics

**Figure 1.**
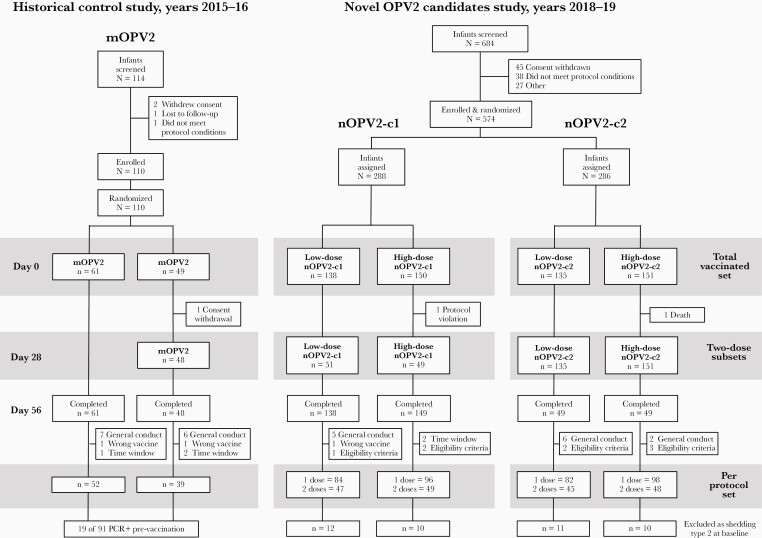
Flowcharts of the 2 studies: the historical control study with monovalent poliovirus type 2 vaccine and later study with both novel poliovirus type 2 vaccine candidates. Abbreviations: mOPV2, monovalent poliovirus type 2 vaccine; nOPV2, novel poliovirus type 2 vaccine; OPV2, oral poliovirus vaccine; PCR, polymerase chain reaction.

The 684 infants enrolled in both studies had similar demographic characteristics across groups (Table 1). In the historical control study, 110 of 114 enrolled infants received a first dose of mOPV2 and 48 received a second dose (Figure 1). In the nOPV2 study, all 574 enrolled infants received a first dose and 199 received a second dose.

**Table 1.** Demographics of the Enrolled Infant Populations in the 2 Studies

Characteristic	Historical Control Study	nOPV2 Study			
	mOPV2	nOPV2-c1		nOPV2-c2	
	Standard Dose	Low Dose	High Dose	Low Dose	High Dose
	(n = 110)	(n = 138)	(n = 150)	(n = 135)	(n = 151)
Age, wk, mean (SD)	19.0 (0.9)	18.7 (1.0)	18.5 (0.8)	18.6 (0.9)	18.4 (0.8)
Male sex, No. (%)	61 (55)	69 (50)	71 (47)	74 (55)	73 (48)
Weight, kg, mean (SD)	7.2 (1.0)	7.2 (0.9)	7.1 (0.8)	7.4 (1.0)	7.2 (0.9)
Race/ethnicity, No. (%)					
Mixed race	108 (98)	135 (98)	147 (98)	132 (98)	151 (100)
Black	2 (2)	0	1 (1)	1 (1)	0
Central American Indian	0	3 (2)	2 (1)	1 (1)	0
Hispanic	0	0	0	0	0
White	0	0	0	1 (1)	0

Abbreviations: mOPV2, monovalent poliovirus type 2 vaccine; nOPV2-c, novel oral poliovirus vaccine candidate; SD, standard deviation.

In the mOPV2 study, 19 of 83 (22.9%) infants were shedding type 2 virus at baseline, probably due to environmental exposure to shed virus prior to OPV2 cessation. In the nOPV2 study, baseline samples from 43 of 380 (11.3%) participants yielded positive PCR results with very low levels of detectable type 2 poliovirus before nOPV2 administration. All participants with any type 2 PCR-positive predose sample were excluded from analysis.

### Extent of Viral Shedding

**Figure 2.**
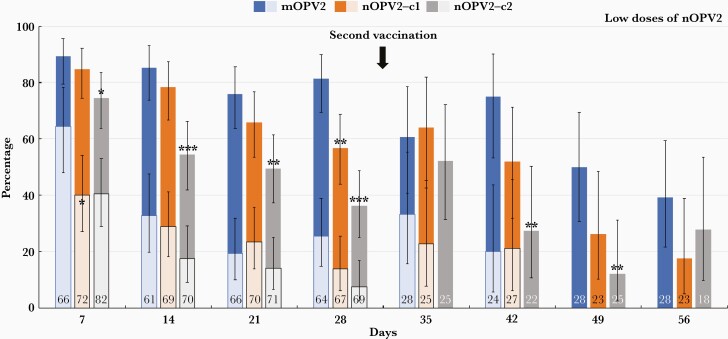
Proportions (with 95% confidence interval) of each study group with polymerase chain reaction–positive stools for poliovirus type 2 (dark bars) or had infective virus (superimposed light bars) at the indicated time-points after first and second low doses of novel poliovirus type 2 vaccine (nOPV2), with the standard monovalent poliovirus type 2 vaccine (mOPV2) for comparison. Numbers in bars indicate numbers of samples. Significant differences vs mOPV2: ∗*P*<.05; ∗∗*P*<.01; ∗∗∗*P*<.001.

**Figure 3.**
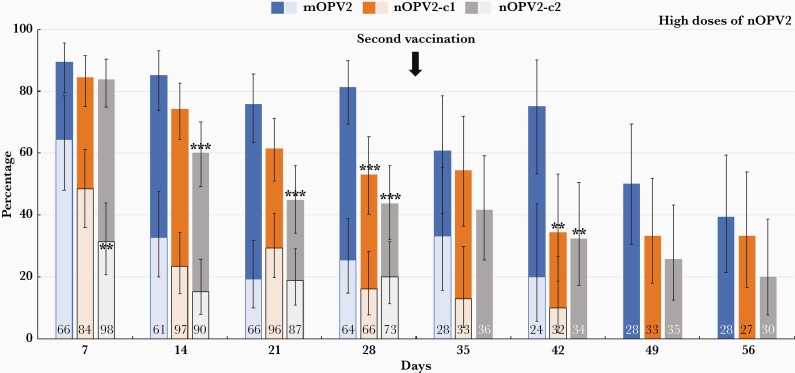
Proportions (with 95% confidence interval) of each study group with polymerase chain reaction–positive stools for poliovirus type 2 (dark bars) or had infective virus (superimposed light bars) at the indicated time-points after first and second high doses of novel poliovirus type 2 vaccine (nOPV2), with the standard monovalent poliovirus type 2 vaccine (mOPV2) for comparison. Numbers in bars are numbers of samples. Significant differences vs mOPV2: ∗*P*<.05; ∗∗*P*<.01; ∗∗∗*P*<.001.

Detection rates of viral RNA (RT-PCR positive) and infectious virus (RT-PCR positive and log_10_ CCID_50_ ≥ 2.75) after first doses of mOPV2 and low and high doses of nOPV2 vaccine candidates are shown in Figures 2 and 3, respectively. Seven days after receiving mOPV2, 89.4% were shedding viral RNA and 81.3% were still PCR positive at day 28. The proportion with measurable infectious virus was 64.3% at day 7, which progressively declined to 25.5% by day 28. Corresponding PCR-positive rates following low-dose nOPV2-c1 and nOPV2-c2 were 84.7% and 74.4% at day 7, which declined to 56.7% and 36.2%, respectively, by day 28 (Figure 2). Proportions with infectious virus were lower: 40.0% and 40.6% at day 7, declining to 13.8% and 7.6% in low-dose nOPV2-c1 and nOPV2-c2 groups, respectively, by day 28. For high-dose nOPV2 groups, the pattern was similar (Figure 3), as PCR-positivity rates declined from 84.5% and 83.7% for nOPV2-c1 and nOPV2-c2 at day 7, to 53.0% and 43.8% at day 28. The infectious virus rates decreased from 48.5% and 31.3% at day 7 to 16.1% and 20.0% by day 28 for high-dose nOPV2 candidates 1 and 2, respectively.

After a second mOPV2 dose, the rate of PCR-positive stools continued to follow the downward course observed after the first dose, reaching 39.3% at day 56. A slight increase in the proportion with infective virus to 33.3% at day 35, 7 days after administration of the second dose, rapidly declined to 20% by day 42; infectious virus was not measured at the 2 later time-points. After second low- and high-doses of nOPV2 candidates, PCR-positivity rates briefly increased at day 35 before declining; at day 56 rates were 17.4% and 33.3% for low- and high-dose nOPV2-c1, and 27.8% and 20.0% for low- and high-dose nOPV2-c2. Culture-positive stools were detected in 22.7% and 21.1% of the low-dose nOPV2-c1 group, and 12.9% and 10.0% of the high-dose nOPV2-c1 group at days 35 and 42. Infectious virus was not measured after second doses of the nOPV2-c2 candidate, following the selection of nOPV2-c1 for EUL submission.

The median log_10_ CCID_50_/g was generally near or below the lower limit of quantitation (LLOQ), and similar across groups at day 7 (≤2.75–2.78, all groups) and significantly lower for all nOPV2 groups at day 28 (0.0–≤2.75 for nOPV2 groups, ≤2.75 for mOPV2). Median SIEs for low-dose (2.73) and high-dose (1.77) nOPV2-c1 were not significantly different from mOPV2 (2.75) following the first dose. The median SIE was significantly lower for low-dose nOPV2-c2 (≤2.75, *P*=.0172), but not for high-dose nOPV2-c2 (≤2.75, *P*=.0607). The AUC was not different between mOPV2 and nOPV2 groups.

### Time to Cessation of Viral Shedding

Median time to shedding cessation exceeded 28 days for all groups following the first dose. Following 1 mOPV2 dose, 87.8% of infants had not yet met the PCR definition of cessation of shedding at day 28, with similar proportions of 81.1% and 77.3% not yet meeting the cessation endpoint after low- and high-dose nOPV2-c1, and significantly lower proportions of 63.4% and 65.4% after low- and high-dose nOPV2-c2, respectively. Time to culture negativity results mirrored PCR results, with overall time to cessation substantially earlier in the nOPV2-c2 groups; medians ranged from 7 days for nOPV2-c2 groups to 12–13 days for mOPV2 and both nOPV2-c1 groups (Table 2). Time to transmission negativity was not different between mOPV2 and nOPV2 groups, with common median of 1 day.

**Table 2.** Fecal Shedding of Poliovirus Type 2 After the First Vaccination With Monovalent Poliovirus Type 2 Vaccine in the Historical Study or Novel Oral Poliovirus Type 2 Candidates in the Subsequent Study

Poliovirus Type	mOPV2^a^	nOPV2-c1				nOPV2-c2			
		Low Dose		High Dose		Low Dose		High Dose	
	(n = 89)	(n = 125)	Δ vs mOPV2	(n = 140)	Δ vs mOPV2	(n = 124)	Δ vs mOPV2	(n = 141)	Δ vs mOPV2
PCR^+^/CCID_50_^+^, No. (%) (95% CI)	69 (77.5) (67.4–85.7)	96 (77.4) (69.0–84.4)	–0.1 (–11.3 to 11.7)	110 (78.6) (70.8–85.1)	1.0 (–9.6 to 12.5)	83 (67.5) (58.4–75.6)	–10.0 (–21.7 to 2.3)	104 (74.8) (66.8–81.8)	–2.7 (–13.7 to 9.0)
	…	…	*P*=1.0000	…	*P*=.8707	…	*P*=.1238	…	*P*=.7513
Days to peak shedding, median (95% CI)	6.0 (5.0–7.0)	6.0 (5.0–7.0)	…	6.0 (5.0–7.0)	…	6.0 (5.0–7.0)	…	5.0 (4.0–6.0)	…
Max. peak titer, log_10_CCID_50_, median (95% CI)	3.16 (3.03–3.75)	3.77 (3.13–4.19)	0.61 (–.23 to 1.05)	3.25 (3.00–3.61)	0.09 (–.53 to .53)	3.44 (3.03–3.75)	0.28 (–.44 to .59)	3.06 (2.91–3.41)	–0.09 (–.69 to .31)
	…	…	*P*=.3571	…	*P*=.7369	…	*P*=.7150	…	*P*=.4612
Log_10_ AUC (95% CI)	n=18	n=42	0.26 (–.11 to .79)	n=49	–0.03 (–.37 to .16)	n=39	0.04 (–.53 to .26)	n=46	–0.08 (–.52 to .37)
	4.00 (3.94–4.30)	4.26 (4.07–4.77)	*P*=.2656	3.97 (3.79–4.14)	*P*=.7772	4.04 (3.63–4.25)	*P*=.8704	3.92 (3.60–4.46)	*P*=.7369
SIE, median (95% CI)	n=25	n=345	–0.02 (–.70 to .67)	n=50	–0.98 (–1.37 to –.02)	n=40	–1.37 (–2.07 to –.66)	n=57	–1.37 (–2.05 to –.66)
	2.75 (2.07–2.77)	2.73 (2.06–2.77)	*P*=.8683	1.77 (1.38–2.16)	*P*=.1605	1.38 (.69–1.80)	*P*=.0172	1.38 (.70–1.68)	*P*=.0607
TTCN, d, median (95% CI)	13 (8–20)	13 (8–16)	*P*=.4853	12 (9–20)	*P*=.6760	7 (6–9)	*P*=.0003	7 (6–8)	*P*=.0118
TTTN, d, median (95% CI)	1 (1–5)	1 (NC–NC)	*P*=.3645	1 (NC–NC)	*P*=.3956	1 (NC–NC)	*P*=.1765	1 (NC–NC)	*P*=.7307

*P* values by χ^2^ test.

Abbreviations: AUC, area under the curve; CCID_50_, 50% cell culture infective dose; CI, confidence interval; mOPV2, monovalent oral poliovirus type 2 vaccine; NC,not computable; nOPV2-c, novel oral poliovirus vaccine candidate; PCR, polymerase chain reaction; SIE, shedding index endpoint; TTCN, time to culture negative; TTTN, time to transmission negative.

Standard dose of mOPV2.

### Poliovirus Types 1 and 3

Shedding of poliovirus types 1 and 3, presumably from the last bOPV vaccination received 4 weeks earlier, was also detectable before and after OPV2 administration (Table 3, Supplementary Figures 1 and 2). In the historical study, 72 of 91 (79.1%) infants were PCR-positive for poliovirus type 1 at any time after receiving 1 dose of mOPV2. PCR-positive stools for poliovirus type 1 were obtained from 179 of 265 (67.5%) infants who received their first doses of nOPV2-c1, and 180 of 265 (67.9%) infants who received first doses of nOPV2-c2. Type 3 poliovirus was shed by 65.6% to 75.7% of the groups within 28 days of an OPV2 vaccination.

**Table 3.** Fecal Shedding of Poliovirus Types 1 and 3 Within 28 Days of the First and Second Vaccinations in the Historical (Monovalent Oral Poliovirus Type 2 Vaccine) and Later Novel Oral Poliovirus Vaccine Candidate Studies

Poliovirus Type	mOPV2^a^ Group	nOPV2-c1 Groups				nOPV2-c2 Groups			
		Low Dose	Versus mOPV2	High Dose	Versus mOPV2	Low Dose	Versus mOPV2	High Dose	Versus mOPV2
Type 1 poliovirus, days 1–28 after first dose									
No.	91	125	…	140	…	124	…	141	…
PCR^+^, No. (%)	72 (79.1)	78 (62.4)	–16.7 (–28.3 to –4.4)	101 (72.1)	–7.0 (–17.8 to 4.7)	83 (66.9)	–12.2 (–23.6 to –.0)	97 (68.8)	–10.3 (–21.3 to 1.5)
(95% CI)	(69.3–86.9)	(53.3–70.9)	*P*=.0108	(63.9–79.4)	*P*=.2778	(57.9–75.1)	*P*=.0644	(60.5–76.3)	*P*=.0972
Type 1 poliovirus, days 29–56 (after second dose)									
No.	37	39	…	46	…	39	…	47	…
PCR^+^, No. (%)	21 (56.8)	16 (41.0)	–15.7 (–36.8 to 6.8)	21 (45.7)	–11.1 (–31.7 to 10.5)	19 (48.7)	–8.0 (–29.6 to 14.3)	17 (36.2)	–20.6 (–40.4 to 1.0)
(95% CI)	(39.5–72.9)	(25.6–57.9)	*P*=.2509	(30.9–61.0)	*P*=.3795	(32.4–65.2)	*P*=.5010	(22.7–51.5)	*P*=.0783
Type 3 poliovirus, days 1–28 after first dose									
No.	91	125	…	140	…	124	…	141	…
PCR^+^, No. (%)	68 (74.7)	82 (65.6)	–9.1 (–21.0 to 3.4)	106 (75.7)	1.0 (–10.1 to 12.8)	89 (71.8)	–3.0 (–14.6 to 9.3)	102 (72.3)	–2.4 (–13.6 to 9.6)
(95% CI)	(64.5–83.3)	(56.6–73.9)	*P*=.1788	(67.8–82.6)	*P*=.8770	(63.0–79.5)	*P*=.6448	(64.2–79.5)	*P*=.7620
Type 3 poliovirus, days 29–56 (after second dose)									
No.	37	39	…	46	…	39	…	47	…
PCR^+^, No. (%)	15 (40.5)	18 (46.2)	5.6 (–16.6 to 27.2)	21 (45.7)	5.1 (–16.3 to 25.8)	16 (41.0)	0.5 (–21.3 to 22.2)	15 (31.9)	–8.6 (–29.0 to 11.9)
(95% CI)	(24.8–57.9)	(30.1–62.8)	*P*=.6503	(30.9–61.0)	*P*=.6628	(25.6–57.9)	*P*=1.0000	(19.1–47.1)	*P*=.4936

Abbreviations: CI, confidence interval; mOPV2, monovalent oral poliovirus type 2 vaccine; nOPV2-c, novel oral poliovirus vaccine candidate; PCR, polymerase chain reaction.

mOPV2 standard dose administered in historical control study.

## DISCUSSION

nOPV2 is considered a critically important tool in the final phases of polio eradication, introduced in outbreak response since March 2021 under WHO EUL to interrupt cVDPV2 transmission [19]. Both novel nOPV2 candidates had comparable safety, tolerability, and immunogenicity as licensed Sabin mOPV2 vaccine [13]. We now report the complete shedding characteristics of mOPV2 and both nOPV2 candidates from those trials. nOPV2 candidates were administered in high and low dosages to encompass the range of the live virus anticipated from release (high dose) to end of shelf-life (low dose) in practical use. Our results indicate that nOPV2 is unlikely to be shed at a higher rate or in greater quantities than mOPV2, and nOPV2 shedding may actually be lower and cease earlier in bOPV/IPV-vaccinated infants. Estimated rates of shedding measured as viral RNA and infectious virus were either similar or more commonly lower for nOPV2 than mOPV2 across postvaccination sampling days, for both dosages. This would be anticipated to lead to lower levels of more stable shed virus in the environment, decreasing the potential for new cVDPV2 outbreaks.

There were no statistically significant detectable differences between mOPV2 and nOPV2 for quantitative peak viral shedding (CCID_50_/g), AUC or shedding index endpoint, except for low-dose nOPV2-c2 SIE. There was a consistent trend for rates to be lower for nOPV2 groups compared with mOPV2 across the sampling days. For nOPV2-c1, the time to cessation (PCR) endpoint was not different from mOPV2 although, as previously reported, the proportion shedding on day 28 was significantly lower for both nOPV2 candidates than mOPV2. Time to achieve PCR and culture negativity was significantly shorter than mOPV2 for both low- and high-doses of nOPV2-c2, but not for nOPV2-c1; times to transmission negative were common across groups. Taken together, these results show that viral shedding of mOPV2 and nOPV2 was similar in the first week after vaccination, but viral shedding more distal to vaccination may be lower for nOPV2 compared with mOPV2. Shedding rates were lower after second doses, indicating that first vaccinations of both mOPV2 and each of the nOPV2 candidates induced intestinal immunity. In all 5 groups some infants were still shedding at day 56, 28 days after their second vaccination, the highest proportion being after a second mOPV2 dose (39.3%), with a nonsignificant trend for lower proportions after second low or high doses of nOPV2-c1 (17.4% and 33.3%) or nOPV2-c2 (27.8% and 20.0%).

It was notable that infants were also shedding poliovirus types 1 and 3; >62% had evidence of type 1 RNA and >66% of type 3 RNA, consistent with administration of 3 bOPV doses at least 4 weeks before shedding assessment began.

Baseline type 2 positivity in 29% of the mOPV2 group infants could be explained by the routine use of OPV containing type 2 during the historical control study. However, the weak indication of nOPV2 viruses in stools of 11% of infants before they received an nOPV2 candidate in the nOPV2 study was unexpected. Sequencing these type 2 positive samples showed they were from both nOPV2 candidates and not the Sabin vaccine that had been withdrawn for routine use by this time. There are several indications that this was due to low-level sample contamination during processing of stool samples, leading to aberrant laboratory results due to the high sensitivity of the RT-PCR assays. These include the first nOPV2-positive samples being obtained within 8 days of the first vaccination with low-dose nOPV2-c1), there being no geographic relationship between vaccine use and baseline positive infants, and environmental surveillance of the study area did not find any type 2 positive samples [20]. Furthermore, most positive samples were nOPV2-c1 despite the simultaneous use of nOPV2-c2. Several infants were baseline positive for the candidate they did not receive (2 nOPV2-c1 infants were PCR-positive for nOPV2-c2 at baseline, 15 nOPV2-c2 infants were PCR positive for nOPV2-c1 at baseline), and except for 1 case their postvaccination stools were positive only for their assigned candidate. Importantly, with 1 exception, none of those with baseline type 2 positive samples had values above the infectivity assay LLOQ; 1 sample with a weak positive PCR signal was just above the LLOQ for infectivity.

Removing subjects with baseline positivity from the analysis was prespecified, a conservative approach ensuring evaluation of vaccine virus shedding, unencumbered by potential for OPV2 exposure near the time of first vaccination. The analysis is therefore expected to be robust and to accurately reflect the overall trend in results. However, as the mOPV2 study was conducted prior to cessation, it cannot be determined whether prevaccination type 2 positive stool samples were due to environmental exposure or potential sample contamination.

The main limitation of this study was the limited comparability given the time gap between the historical control and nOPV2 cohorts. Knowing direct comparison of mOPV2 and nOPV2 vaccines was not possible, we specifically designed and performed the historical control study using study centers in the same area, with the same principal investigator, and common protocols, laboratory, and laboratory assessments, etc, to minimize possible confounding factors in the comparisons of the nOPV2 candidates with mOPV2.

The improved genetic stability of nOPV2 candidates is intended to reduce the likelihood of losing key attenuations and reversion to neurovirulence, while maintaining acceptable safety and immunogenicity characteristics. Fecal viral shedding is the primary means of transmission of the reversion-prone Sabin 2 vaccine strain, which in rare cases leads to cVDPV2 but is consequential to the beneficial replication necessary to generate intestinal immunity. Mucosal immunity is induced in response to OPV, and this has an impact on the fecal shedding of the shedding of the virus [21]. While humoral immunity induced by IPV has been shown to have little impact on intestinal immunity [22], we have previously demonstrated that a single dose of mOPV2 was sufficient to induce intestinal immunity in IPV-vaccinated children [23]. Viral shedding has secondary benefits of exposing those unreached by vaccination efforts to virus, potentially providing passive immunization. Our results indicating similar levels of viral shedding to mOPV2 soon after vaccination with nOPV2 candidates, but with generally lower viral shedding more distal to vaccination, offer encouraging signs that the vaccines are replicating in sufficient quantity and duration to generate an intestinal immune response, but are not shed in significantly greater quantities than mOPV2. Assuming similar infectious doses to Sabin 2 implies similar or potentially lower transmissibility. Further evaluation of the genetic stability of shed virus, necessary to further inform policy on the use of nOPV2, will be reported separately. Viral shedding will be further evaluated in ongoing phase 2 studies in Bangladesh with nOPV2-c1 in poliovirus-naive newborns (ClinicalTrials.gov identifier NCT04693286) and concomitant administration of nOPV2-c1 with bOPV in infants (ClinicalTrials.gov identifier NCT04579510), and an ongoing phase 3 study of the safety of nOPV2 in The Gambia (WHO PACTR202010705577776). Our data will inform decisions by countries considering using nOPV2-c1 under the WHO EUL, licensure and prequalification considerations, and modeling efforts aimed at estimating transmissibility.

## Supplementary Data

Supplementary materials are available at *The Journal of Infectious Diseases* online. Supplementary materials consist of data provided by the author that are published to benefit the reader. The posted materials are not copyedited. The contents of all supplementary data are the sole responsibility of the authors. Questions or messages regarding errors should be addressed to the author.

jiab507_suppl_Supplementary_Materials_1

## Notes

***Acknowledgments.*** The authors are grateful to all parents and guardians who allowed their children to participate in this study. We wish to thank the team at the Centers for Disease Control and Prevention (CDC) who performed all viral analyses, including Demetrius Mathis, Ling Wei, Brittani Brown, and Lizheng Guo. We also thank Assign Data Management and Biostatistics GmbH (Innsbruck, Austria) who did the statistical analyses under the supervision of Dr Chris Gast, PATH, and Carrie Trujillo for her role in coordination. In Panama the Ministry of Health and Expanded Program of Immunization authorities discussed the study protocol and provided guidance on implementing the study immunization schedule. The data and safety monitoring board was comprised of Professor Dr Fred Zepp (Chairman), Professor Lilly Yin Weckx, Professor Jarir At Thobari, and Dr Jean Fernandez, with Dr Patrick Zuber and Dr Carolina Danavaro as observers. The manuscript was drafted and managed by Keith Veitch (keithveitch communications, Amsterdam, the Netherlands).

***Financial support.*** This work was sponsored by Fighting Infectious Diseases in Emerging Countries (FIDEC), with grant funding by the Bill & Melinda Gates Foundation (grant number OPP1150472).

***Dedication.*** On behalf of my co-authors, I (A.S.B.) would like to dedicate this work to the loving memory of our colleague and public health professional N. S. B. from Bio Farma, who passed away while this manuscript was being finalized.
