# Supplementary material for: Fecal Shedding of 2 Novel Live Attenuated Oral Poliovirus Type 2 Vaccine Candidates by Healthy Infants Administered Bivalent Oral Poliovirus Vaccine/Inactivated Poliovirus Vaccine: 2 Randomized Clinical Trials
Source: J Infect Dis. 2021 Oct 5;226(5):852–61. doi: 10.1093/infdis/jiab507 (PMC9470102; doi:10.1093/infdis/jiab507)
Supplement: jiab507_suppl_Supplementary_Materials_1 [file jiab507_suppl_supplementary_materials_1.docx]

**Fecal shedding of two novel live attenuated oral poliovirus type 2 vaccines candidates by healthy bOPV/IPV-vaccinated infants: two randomized clinical trials**

**Christopher Gast et al**

**Supplementary material**

**Supplementary Figure 1.** Proportions of infants with PCR+ stools for poliovirus type 1

**Supplementary Figure 2.** Proportions of infants with PCR+ stools for poliovirus type 3

**Supplementary Figure 1.** Proportions (with 95% CI) of each study group with PCR+ stools for poliovirus type 1 at the indicated time-points after first and second low- (upper panel) or high-doses (upper panel) of the nOPV2 candidates, with the standard mOPV2 for comparison.

**Supplementary Figure 2.** Proportions (with 95% CI) of each study group with PCR+ stools for poliovirus type 3 at the indicated time-points after first and second low- (upper panel) or high-doses (upper panel) of the nOPV2 candidates, with the standard mOPV2 for comparison.
